# Believing and Perceiving: Authorship Belief Modulates Sensory Attenuation

**DOI:** 10.1371/journal.pone.0037959

**Published:** 2012-05-29

**Authors:** Andrea Desantis, Carmen Weiss, Simone Schütz-Bosbach, Florian Waszak

**Affiliations:** 1 Université Paris Descartes, Sorbonne Paris Cité, Paris, France; 2 CNRS (Laboratoire Psychologie de la Perception, UMR 8158), Paris, France; 3 Max Planck Institute for Human Cognitive and Brain Sciences, Leipzig, Germany; 4 Ecole des Hautes Etudes en Sciences Sociales, Paris, France; 5 CNRS (Institut Jean Nicod UMR 8129), Paris, France; Royal Holloway, University of London, United Kingdom

## Abstract

Sensory attenuation refers to the observation that self-generated stimuli are attenuated, both in terms of their phenomenology and their cortical response compared to the same stimuli when generated externally. Accordingly, it has been assumed that sensory attenuation might help individuals to determine whether a sensory event was caused by themselves or not. In the present study, we investigated whether this dependency is reciprocal, namely whether sensory attenuation is modulated by prior beliefs of authorship. Participants had to judge the loudness of auditory effects that they believed were either self-generated or triggered by another person. However, in reality, the sounds were always triggered by the participants' actions. Participants perceived the tones' loudness attenuated when they believed that the sounds were self-generated compared to when they believed that they were generated by another person. Sensory attenuation is considered to contribute to the emergence of people's belief of authorship. Our results suggest that sensory attenuation is also a consequence of prior belief about the causal link between an action and a sensory change in the environment.

## Introduction

Many movements result in sensory consequences similar to sensory input elicited by external events. However, several studies observed that self- and externally generated sensory signals are processed differently. Notably, self-generated stimuli are attenuated both in terms of their phenomenology [1]–[3] and their cortical response [4], [5] compared to the same stimuli externally generated. It has often been assumed that sensory attenuation emerges from internal forward models that predict the sensory consequences of an ongoing action [3], [6]–[8]. In line with this idea, several studies show that sensory attenuation varies as a function the predictability of the perceptual action consequences [3], [6], [9] and that it is genuinely a result of motor prediction and not of prediction in general [1].

The phenomenon of sensory attenuation has been considered to enhance the ability to differentiate externally triggered sensory changes from self-produced action effects [10]. Moreover, attenuation is also thought to be an important perceptual cue contributing to the emergence of our belief of authorship, i.e., the belief of being the cause of a sensory change in the environment [2], [11]. The link between sensory attenuation and beliefs of authorship has been emphasized in the study of schizophrenia patients showing symptoms such as delusions of control and thought insertion. Specifically, several studies show that these patients fail to attenuate responses to the sensory consequences of their actions and speech [12]–[15], thus leading to difficulties in distinguishing internally from externally generated stimuli [15], [16].

However, the relationship between sensory attenuation and belief of authorship must not necessarily be a one-way road from sensory attenuation to belief of authorship. It is also possible that authorship beliefs modulate sensory attenuation. Note that the contrasts that have usually been used to assess sensory attenuation compared conditions in which sensory events were either externally triggered or produced by a voluntary action. These conditions differ in motor predictive processes and the causal link between action and effect (prior belief of authorship) at the same time [17]. The present study, using self-produced stimuli in all conditions, investigates whether belief of authorship alone results in sensory attenuation, i.e., whether authorship belief is a driving force behind sensory attenuation instead of merely being inferred from perceived attenuation.

An influence of the belief of authorship on perception has recently been observed in experiments on intentional binding [18]–[20], i.e., the finding that when a voluntary action produces a sensory event, action and outcome are perceived as closer together in time [21]. This phenomenon has been interpreted to be based on the same predictive motor mechanisms discussed above [22], [23]. However, two recent studies [24], [25] suggest that sensory attenuation and intentional binding are not based on the same mechanisms. Thus, it is still unknown whether prior authorship beliefs influence sensory attenuation in the same way.

To shed further light on this issue we assessed the perceived loudness of auditory stimuli [2], [9], [26], [27] in a social setting that allowed for the manipulation of participants' prior belief of authorship. Specifically, participants were led to believe that a sound was either triggered by themselves or by someone else, although, in reality, the sound was always triggered by participants themselves. To foreshadow the results, we found the perceived loudness of the sound to be attenuated when participants believed that the sound was triggered by their own action, compared to when they believed that it was triggered by another agent. We will discuss the implications of the finding in the discussion.

## Materials and Methods

### Participants

Twelve subjects (average age 22.7 years, *SD* = 3.5 years) participated in the experiment for an allowance of € 10/h. All had normal or corrected-to-normal vision and hearing and were naïve as to the hypothesis under investigation. They all gave written informed consent. The study was conducted in accordance with the Declaration of Helsinki and was approved by the local Ethics Committee.

### Material

Stimulus presentation and data acquisition were conducted using the psychophysics Toolbox [28], [29] for Matlab 7.5.0 running on a PC computer connected to two 19-in. 85 Hz CRT monitors. Auditory stimuli were presented via a pair of headphones.

### Stimuli and procedure

Participants were informed that the experimenter takes part in the experiment as a second participant, as the real second participant has canceled. The experiment consisted of two phases: A belief implementation phase and a test phase. In both phases we used two monitors (one in front of the participant, and the other in front of the experimenter) and one keyboard connected to the same PC.

#### Belief implementation phase

Participants carried out two conditions in separate blocks of 100 trials each. Block order was counterbalanced across participants. In the *self* condition the participant's name was displayed on the center of both screens. Participants were required to execute right index finger key presses at a self-paced rate. In the *other* condition the experimenter's name was displayed on the screens. The participant observed the experimenter executing self-paced key-presses. Both the participants' and the experimenter's key press actions were followed by a 1000 Hz tone after an interval (stimulus onset asynchrony; SOA) of 150, 300 or 450 ms. The tone was presented for 100 ms with a pressure level of 74 dB. The probability that the tone occurred after one of the three intervals was set to .7 for the 300 ms SOA .15 for the 150 ms SOA and .15 for the 450 ms. At the beginning of each block the participant was shown that only the key press of the person whose name was displayed on the screens could trigger the tone. The aim of this phase was to make the participant adopt two contextual beliefs: 1) if my name is displayed, the tone follows my action; 2) if the experimenter's name is displayed, the tone follows his action.

Thereafter, participants ran another belief implementation block that was more similar to the test phase. Here, a clock-face and a clock-hand (rotating with a period of 3 sec.) were displayed on the two screens. The clock-face included a shaded area. The size of the shaded area was 60° of the clock-face; its location was randomly chosen. The participants were shown that only the person whose name was displayed on the screens could trigger the tone by executing a key press while the clock-hand passed through the shaded area (no tone was delivered if that person executed a key press outside the shaded area). The participant's or experimenter's name was displayed either below or above the clock-face (counterbalanced across participants). Participant ran 10 trials per condition (self and other). The second belief implementation phase was meant to familiarize participants with the task procedure of the test phase.

#### Test phase

The participant and experimenter were separated by a card board. Both saw the same clock-face, clock-hand and a randomly located shaded area on the screen (see above). Participants ran two conditions: a *self* and an *other* condition. In these conditions either the participant's (*self* condition) or the experimenter's name (*other* condition) was displayed on the screens, in order to make the participants believe that, in the given trial, the tone would either be triggered by their own or the experimenter's action. In both conditions, the participant and the experimenter had to execute a key press when the clock-hand passed through the shaded area for the first time. Participants wore sound isolating headphones preventing them to hear and being distracted by the experimenter's key-presses. Furthermore, the experimenter was instructed to avoid any noisy key-presses. Importantly, contrary to the belief implementation phase, the tone was always (expect in some rare case, see below) triggered by the participant's key press regardless of the name displayed on the screens. The tone appeared after one of three SOAs (150, 300 or 450 ms) with the same probability we used in the belief implementation phase. We used three SOAs to introduce variability between the participants' action and the occurrence of the sound. This was meant to prevent the participants from realizing that they triggered the tone in both belief conditions. We chose these three interval probabilities to calibrate internal forward model to predict in particular the tone at 300 ms SOA. Data analysis focused on the 300 ms SOA. The other SOAs were just used to induce the beliefs. Moreover, in order to strengthen the participants' authorship beliefs, the experimenter deactivated the participants' key twice in the *other* condition and triggered the tone himself (mostly during the second rotation of the hand).

**Figure 1:**
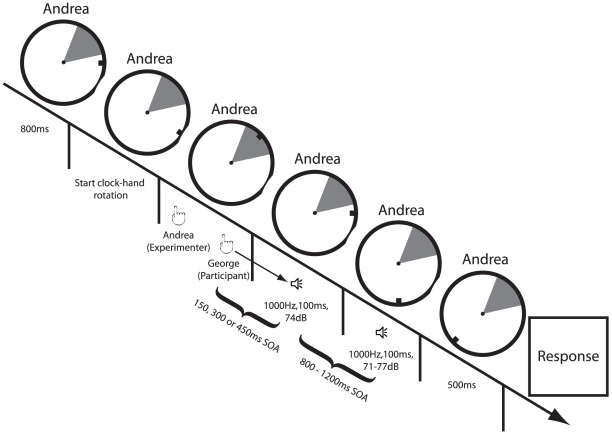
Schematic representation of an experimental trial. At the beginning of each trial of both belief conditions the participant's name (self condition) or experimenter's name (other condition) blinked for 800 ms. Then, the name stopped to blink and the clock-hand started to rotate. Participant and experimenter had to execute a key-press when the clock-hand first passed through the shaded area. The participant always triggered the sound in both belief conditions after one of three possible SOAs (150, 300 or 450 ms). After a random interval (between 800–1200 ms) from the occurrence of the first tone a second tone occurred. Finally, 500 ms after the occurrence of the second tone the clock-hand stopped and both participant and experimenter answered the question: “Which one of the two tones was louder?”

To assess the influence of prior belief on participants loudness perception we used a comparison task procedure that have been previously used to show sensory attenuation of self-generated sensory event [2], [26], [27]. At the end of each trial (see Figure 1) participants and experimenter were required to compare the loudness of the standard tone (1000 Hz, 100 ms, 74 dB) generated by the key press with a comparison tone of the same frequency and duration but varying in magnitude. The comparison tone was presented after a random interval of 800–1200 ms. Its sound pressure level varied randomly between 71 and 77 dB in 1 dB steps. The participants judged which of the two tones (the standard tone or comparison tone) was louder by pressing with their left hand one of two keys. At the end of the experiment participants had to answer yes or no to the following questions: 1) “Did you believe that whenever your name was displayed on the screens you and not the other participant triggered the first tone?”; 2) “Did you believe that whenever the other participant's name was displayed on the screens s/he and not you triggered the first tone?”.

The test phase consisted of 200 trials per belief condition (140 trials with 300 ms SOA, and 30 trials for each of the two other SOAs). For the 300 ms SOAs of both belief conditions each of the seven comparison tone magnitudes was presented 20 times. Each belief condition was presented in 10 mini-blocks of 20 trials. Mini-blocks were presented alternately with block presentation being counterbalanced across subjects.

## Results

As mentioned above data of the 150 and 450 ms SOA were excluded from analysis. The proportion of “second tone louder” responses for the 300 ms SOA was calculated separately for each participant, condition and the seven magnitudes of the comparison tone. Psychometric functions were fitted using the Psignifit Toolbox version 2.5.6 for Matlab (see http://bootstrap-software.org/psignifit/) which implements the maximum-likelihood method described by Wichmann and Hill [30]. Based on each individual function, we calculated the point of subjective equality (PSE) and the just noticeable difference (JND). The PSE, defined as the comparison tone magnitude judged as louder than the first tone on 50% of trials, reflects the perceived loudness intensity of the first tone under the two belief conditions [2], [26]. For instance, if sensory attenuation is stronger in the *self* condition, one would expect a significantly lower PSE value in this condition compared to the *other* condition. The JND, defined as the difference of the comparison tone magnitude judged as louder on 75% and judged as louder on 25% of trials divided by 2, assesses the precision of responses as it reflects the variability of loudness perception. For example, if the self belief manipulation would lead to a general impairment in the amount of available perceptual information (e.g., due to a reduction of attention in this condition) and therefore to an increase of response variability, one would expect a higher JND value in the self condition compared to the other condition. The level of significance of our analysis was set at p<.05 for all statistical tests.

All the participants believed i) that they triggered the sound when their name was displayed on the screen and ii) that the experimenter triggered the sound when his name was displayed. Hence, although in reality the tone was always triggered by the participants' action, they adopted different authorship beliefs in the two conditions.

**Figure 2:**
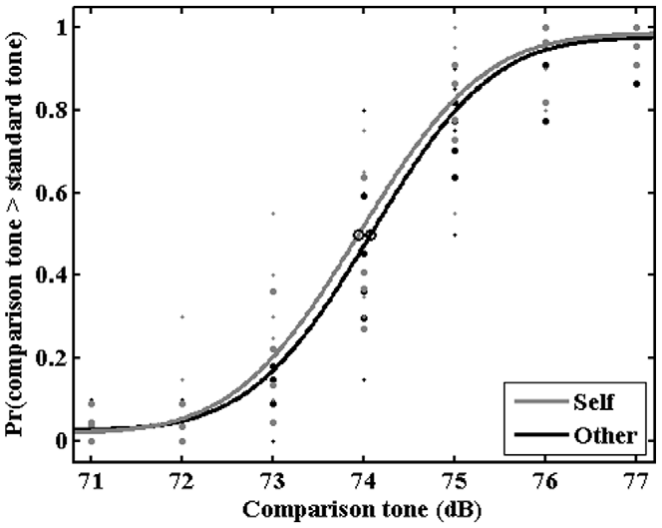
Proportion of “second tone louder” responses for the 300 ms SOA for the self and other condition (averaged across all participants) as a function of the seven comparison tone magnitudes.

PSE values of the *self* and *other* condition were compared using a paired one-tailed t-test. The analysis revealed that PSE values were significantly lower in the *self* condition compared to the *other* condition t(11) = 2.73, p = .010 (Figure 2), indicating a reduction on the perceived intensity of the stimulus in the former compared to the latter.

A paired two-tailed t-test on JND values showed no significant difference between the two conditions t(11) = 0.07, p = .943. This indicates that response variability did not differ in the two belief conditions, suggesting that the effect of attenuation we observed in the self condition compared the other condition was specifically related to a reduced intensity perception of the standard tone (PSE) but not to a general reduction in the amount of available perceptual information (JND).

## Discussion

Self-generated auditory stimuli have largely been observed to be attenuated compared to those externally generated [2], [9], [26], [27], [31]–[33]. However, the contrast of self vs. externally produced stimuli differs in both motor predictive processes and authorship belief. Therefore, in the present study we contrasted self-produced stimuli in two conditions differing only in the participants' authorship belief. Both belief conditions were identical in terms of sensorimotor-based temporal predictability, since, unbeknownst to the participant, it was always the participant who triggered the tone. First of all, note that the fact that our manipulation worked (i.e., we successfully induced different belief of authorship), confirms that authorship is not directly perceived but inferred from different cues as prior knowledge and other context-related factors [34]–[36]. More importantly, we found a reduction of the PSE value when participants believed that their own action triggered the tone compared to when they believed that the other participant's action triggered the tone. Our results, thus, show that differences in authorship belief are sufficient to yield sensory attenuation.

One possible explanation for this result is that participants allocate attentional resources differently in the two conditions. Previous studies have shown that allocating more/less attention to stimuli alters their perceived intensity [37], [38]. However, this explanation seems unlikely to us for two reasons. Firstly, in both conditions participants' attention was likewise oriented to the clock hand. Secondly, Anton-Erxleben et al., [37] (see experiment 3 & 4) showed that in comparison tasks a difference in allocation of attention does not only modulate the perceived intensity of the stimulus (PSE) but also the response variability (reflected by the JND in the present experiment). However, in our experiment the discrimination performance (JND) was identical in the two belief conditions. Thus, it is unlikely that the difference in intensity attenuation (PSE) we observed is due general change in attentional attunement.

The present study demonstrates that differences in authorship belief alone can result in sensory attenuation. However, note that this does not mean to say that internal forward models do not play any role in the emergence of sensory attenuation. In the contrary, we believe that our results can be accounted for in terms of predictive motor processing. Several scenarios are possible. In the most radical one, prior authorship belief determines whether or not the action's consequence is (motor) predicted in the first place. If the sensorimotor system believes that somebody else triggers the upcoming stimulus why should it predict the event? One could argue that, in this case, the system actually makes an invalid prediction, as the prediction might erroneously result in self-attribution of the given stimulation.

A less radical explanation would be that authorship belief influences how reliable the brain considers internal forward models to be. Specifically, depending on whether or not the agent believes to be the cause of an upcoming event, predictive motor signals might get high and low weights, respectively, in a process integrating different cues concerning the self/other distinction of the upcoming stimulus [39]–[41].

One might argue that the effect we observed was partly due to the fact that participants were not completely free to time their actions, since they had to act within the shaded area. Self-initiated action yield stronger activation of action control structures (e.g., the SMA complex) compared to action that are externally triggered [42]. Although several studies observed sensory attenuation in conditions in which participants responded to a go signal [4], [6], [43] (suggesting that internal prediction is still available to the subject), it is possible that weak internal predictive signals (due to the fact that participants were not completely free to time their action) might have made people more susceptible to the influence of external cues such as the prior belief of authorship [39], [44]. Further research is necessary to clarify this issue.

Our findings might also provide important information for the understanding of delusions of control in schizophrenia. Our results suggest that abnormal prior beliefs of authorship may contribute to the emergence of abnormal perceptions. This is in agreement with recent Bayesian integrative accounts of agency which suggest that people's perception of sensory action consequences and their experience of being in control of their actions is based on the combination of prior knowledge and the likelihood obtained from sensory input [13], [39]. Our results might indicate that a globally altered belief of oneself as an agent may lead to an inadequate weighting of different authorship indicators, and may, thus, result in the emergence of abnormal perceptions/beliefs in a specific situation. For instance, abnormal beliefs may result in assigning a particular salience to stimuli that in reality have to be attenuated because they are self-generated. These stimuli might feel strange and externally generated [13], [39].

Sensory attenuation has usually been assessed comparing self-vs. externally generated stimuli. Three recent studies, for example, observed attenuation of self- vs. externally generated auditory stimuli using exactly the same stimuli and procedure as the present study [2], [26], [27]. As mentioned above, this contrast covers, amongst others, motor prediction and authorship belief. The main interest of the present study was to isolate the effect of authorship belief. To do so, we did not necessarily need to include a condition in which stimuli are externally generated. Since the manipulation of authorship belief was rather challenging, we decided to renounce on this type of condition. However, future research could compare the strength of authorship-induced sensory attenuation with the strength of sensory attenuation based on the contrast of external vs. self-produced stimuli to further clarify the different factors contributing to the phenomenon of sensory attenuation.

Finally, we would like to point out one caveat. We (and all other studies using a similar methodology) cannot tell for sure whether our manipulation affected participants' sensitivity or whether it induced a response bias. This is because the PSE may be affected by both response criterion and sensitivity. However, using signal detection theory methodology [45], Cardoso-Leite et al. [1] recently showed that sensory attenuation (at least in the visual domain) is based on decreased sensitivity and not on a shift in response bias. We therefore feel confident that this also holds for our experiment.

In conclusion, sensory attenuation is usually considered to contribute to the emergence of people's belief of authorship. Our experiment indicates that the relationship between perception and belief is reciprocal, i.e., that sensory attenuation is also shaped by prior authorship belief.
